# Emerging Mammarenaviruses in Wildlife: Expanding Host Range and Implications

**DOI:** 10.3390/ani16142263

**Published:** 2026-07-22

**Authors:** Barbara Di Martino, Matteo Carnevale, Lorenzo Corsi, Vittorio Sarchese, Francesco Pellegrini, Camilla Smoglica, Antonio Petrini, Vito Martella, Fulvio Marsilio, Federica Di Profio

**Affiliations:** 1 Department of Veterinary Medicine, Università degli Studi di Teramo, Località Piano D’Accio, 64100 Teramo, Italy; bdimartino@unite.it (B.D.M.); mcarnevale@unite.it (M.C.); lcorsi@unite.it (L.C.); vsarchese@unite.it (V.S.); csmoglica@unite.it (C.S.); fmarsilio@unite.it (F.M.); 2 Department of Veterinary Medicine, Università Aldo Moro di Bari, S.p. per Casamassima Km3, Valenzano, 70010 Bari, Italy; francesco.pellegrini@uniba.it (F.P.); vito.martella@uniba.it (V.M.); 3 Istituto Zooprofilattico Sperimentale Dell’Abruzzo E Molise “G. Caporale”, Campo Boario, 64100 Teramo, Italy; a.petrini@izs.it; 4 Department of Pharmacology and Toxicology, University of Veterinary Medicine, 1078 Budapest, Hungary

**Keywords:** *Arenaviridae*, zoonoses, novel mammarenaviruses, wild animals, non-rodent small mammal hosts, spillover risk

## Abstract

Mammarenaviruses are enveloped, ambisense, single-stranded RNA viruses capable of causing fatal hemorrhagic fevers and severe neurological disorders in humans. Although muroid rodents have historically been recognized as the primary reservoirs for major pathogens like Lassa virus, recent surveillance has revealed a significant expansion of their host range. This review aims to synthesize current global data regarding the epidemiology of mammarenaviruses in conventional reservoirs and the emergence of novel arenaviruses in non-traditional mammalian hosts. To achieve this, we comprehensively analyzed recent molecular and metagenomic surveillance data, evolutionary studies, and epidemiological reports published worldwide. Key discoveries include Wenzhou virus in Asian house shrews, Plateau Pika virus in plateau pikas, and an independent, geographically clustered of hedgehog-associated arenaviruses across Europe. Ultimately, this review underscores the global distribution of these pathogens and the critical need for continued, multi-host surveillance worldwide.

## Simple Summary

Mammarenaviruses are significant zoonotic pathogens long believed to be restricted to muroid rodents. However, recent metagenomic and surveillance studies have challenged this long-standing paradigm by discovering highly divergent mammarenaviruses in non-rodent hosts, specifically within the orders Eulipotyphla (hedgehogs and shrews) and Lagomorpha (pikas). This review synthesizes the epidemiology of mammarenaviruses in rodents, and the latest discoveries concerning the complex ecology of mammarenaviruses in other unconventional mammal hosts.

## 1. Introduction

Mammarenaviruses are important zoonotic pathogens recognizing the muroid rodents as the primary virus natural reservoir. Mammarenaviruses are classified into the genus *Mammarenavirus* within the family *Arenaviridae*, along with the genera *Antennavirus*, *Hartmanivirus*, *Innmovirus* and *Reptarenavirus* [1]. These viruses infect mammals (mammarenaviruses), fish (antennaviruses), snakes (hartmaniviruses and reptarenaviruses), and unknown hosts (innmoviruses).

Several mammarenaviruses including Lassa Virus (LASV), Lujo Virus (LUJV), Junin Virus (JUNV), Machupo Virus (MACV), Guanarito Virus (GTOV), and Chapare Virus (CHAPV), are causative agents of viral hemorrhagic fever (VHFs) in humans, posing significant public health problems in their endemic regions [2].

Traditionally, mammarenaviruses were considered to be restricted to specific rodent reservoirs, following a strict codivergence paradigm resulting from long-term evolutionary adaptation [3,4,5]. A notable historical exception was the Tacaribe Virus (TCRV), isolated from phyllostomid bats (*Artibeus* spp.) [6]. Consequently, the geographical distribution of individual mammarenaviruses was thought to be dictated by the limited range of their cognate hosts, with the sole exception of the Lymphocytic Choriomeningitis Virus (LCMV), which achieved a worldwide spread primarily through its natural host, the common house mouse (*Mus musculus Linnaeus*, 1758, *M. musculus*) [7]. However, the rapid accumulation of recent metagenomic and comparative phylogenetic data has dismantled this strict codivergence model. Some evidence demonstrates that the host range of mammarenaviruses is significantly wider than previously suspected, and that host-switching, rather than codivergence, is the predominant driver of mammarenavirus evolution [8,9,10]. The apparent association of some mammarenavirus species with certain rodent subfamilies appears to be shaped by geographical sympatry rather than phylogenetic relatedness [9,11]. The discovery of highly divergent novel mammarenaviruses in non-rodent small mammals highlights this evolutionary plasticity, revealing a host-range expansion from Rodentia to distinct taxonomic orders such as Eulipotyphla and Lagomorpha [5,12,13,14]. Together, these findings underscore a high potential for cross-species transmission, redefining the ecological boundaries and zoonotic risk assessment of emerging arenaviruses. In this context, it could be hypothesized that high viral genomic adaptability, coupled with close ecological sympatry among small mammals, enables mammarenaviruses to successfully colonize and establish stable, self-sustaining transmission cycles in phylogenetically distant mammalian taxa, rather than resulting in transient, dead-end spillover infections.

This review aims to comprehensively evaluate the expanding host range of mammarenaviruses, with a specific focus on newly identified non-rodent species as potential reservoirs. Understanding these dynamics is of high practical significance for refining veterinary surveillance frameworks, providing actionable insights to update zoonotic risk maps where human–wildlife interfaces overlap, and supporting One Health frameworks in predicting and preventing future spillover events.

## 2. Review Methodology

### 2.1. Literature Search and Study Selection

This structured narrative review analyzes mammarenavirus epidemiology, host associations, and potential zoonotic implications. The literature search primarily targeted evidence of mammarenavirus infection or exposure in non-traditional mammalian hosts, such as non-muroid rodents and non-rodent mammals. Additional targeted searches retrieved information on virus taxonomy, established reservoirs, transmission, and zoonotic potential. The literature search was conducted on 30 March 2026 and updated on 28 June 2026 using PubMed, Scopus, Web of Science Core Collection, CAB Abstracts (CABI Digital Library), and Google Scholar. No restrictions were applied regarding publication year or geographic area, and only English-language publications were included. Search terms comprised Mammarenavirus, Arenaviridae, individual virus names, and terms concerning wildlife, reservoirs, host range, and relevant mammalian taxa.

### 2.2. Eligibility Criteria and Data Synthesis

Peer-reviewed primary studies reporting naturally occurring mammarenavirus infection or exposure were considered eligible. Both initial descriptions of virus–host associations and subsequent studies providing data on prevalence, geographic distribution, genetic diversity, tissue tropism, or epidemiological significance were included. Direct molecular or virological detection was distinguished from serological evidence of prior exposure. Experimental and in vitro studies were included only if they provided relevant information on susceptibility, viral replication, or zoonotic potential. Reviews, taxonomic reports, textbooks, and institutional sources were used solely for contextual information. Relevant information was extracted regarding host species, geographic origin, biological matrices, diagnostic methods, virus identity, and prevalence or population-level circulation. Findings were synthesized narratively and organized by virus, host taxonomy, geographic distribution, and type of evidence. Because of substantial heterogeneity in study design, sampling, diagnostic methods, and outcome reporting, prevalence estimates were not statistically pooled. The PubMed search retrieved 1536 records. Records from all databases were exported to EndNote 21, and duplicates were removed. Titles and abstracts were screened to exclude publications outside the scope of the review, such as studies not addressing mammarenavirus natural host associations, wildlife reservoirs, host range expansion, or such ecological and zoonotic implications. Full texts of potentially relevant publications were assessed against the eligibility criteria. Reference lists of relevant articles were also examined to identify additional studies.

## 3. Aetiology

**Figure 1:**
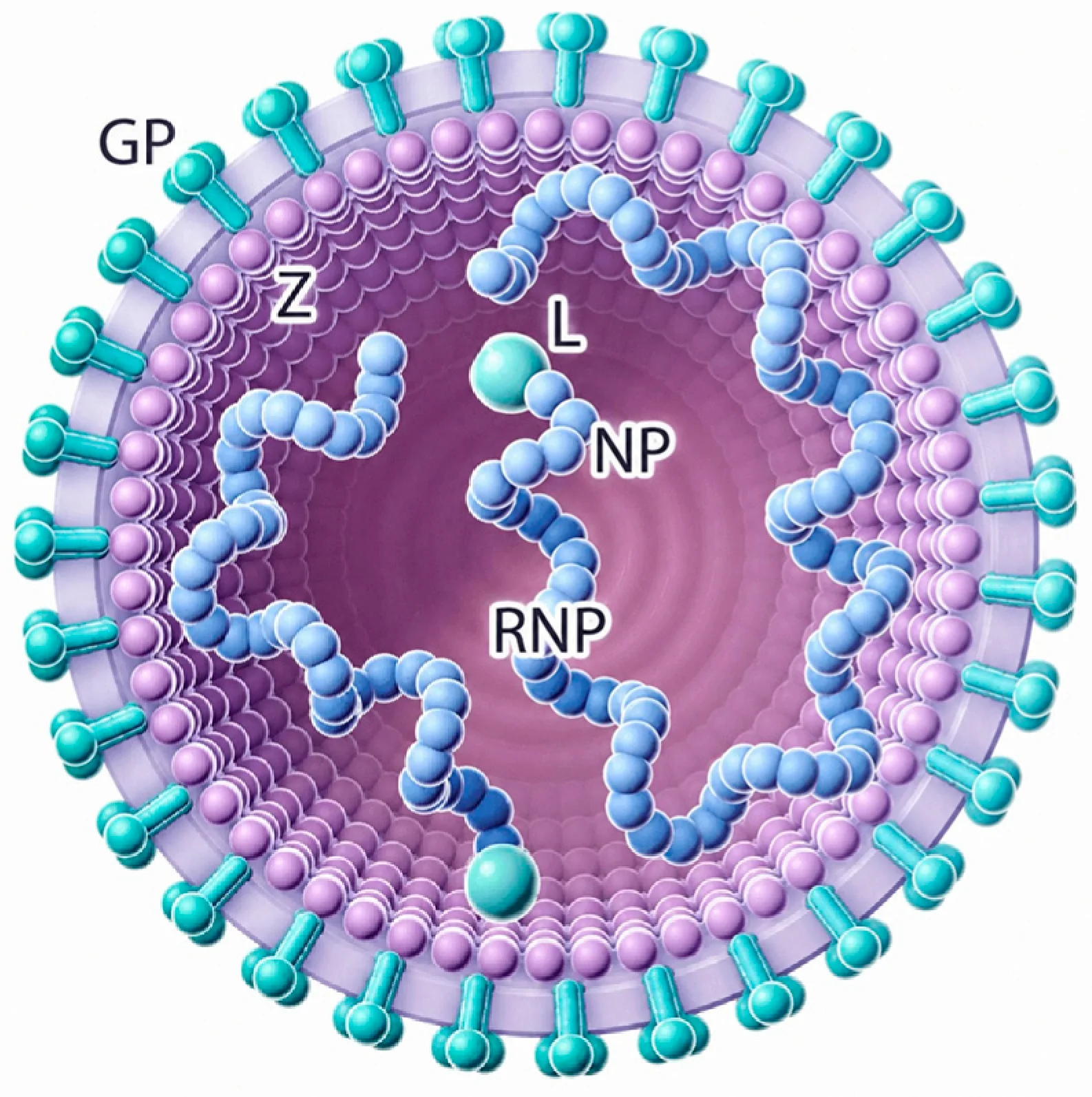
Schematic representation of mammarenavirus structure. Figure was created by using Inkscape, version 1.4.3.

Mammarenaviruses are enveloped, ambisense, single-stranded RNA viruses, spherical or pleomorphic in shape, 50–200 nm in diameter, surrounded by a dense lipid envelope derived from the plasma membrane of the host cell [1,15]. Club-shaped glycoprotein (GP) projections of 8–10 nm in length and 10 nm apart, are evenly distributed on the virion surface [16]. Each GP projection is a trimer of heterodimers comprising the distal subunit GP1, the transmembrane subunit GP2, and a stable signal peptide (SSP) [17,18]. GP1 is responsible for binding to the host cell receptor, GP2 mediates the fusion between the virus envelope and the endosomal membrane at low pH [19,20,21], while the SSP represents an essential component in glycoprotein maturation [22] (Figure 1).

**Figure 2:**
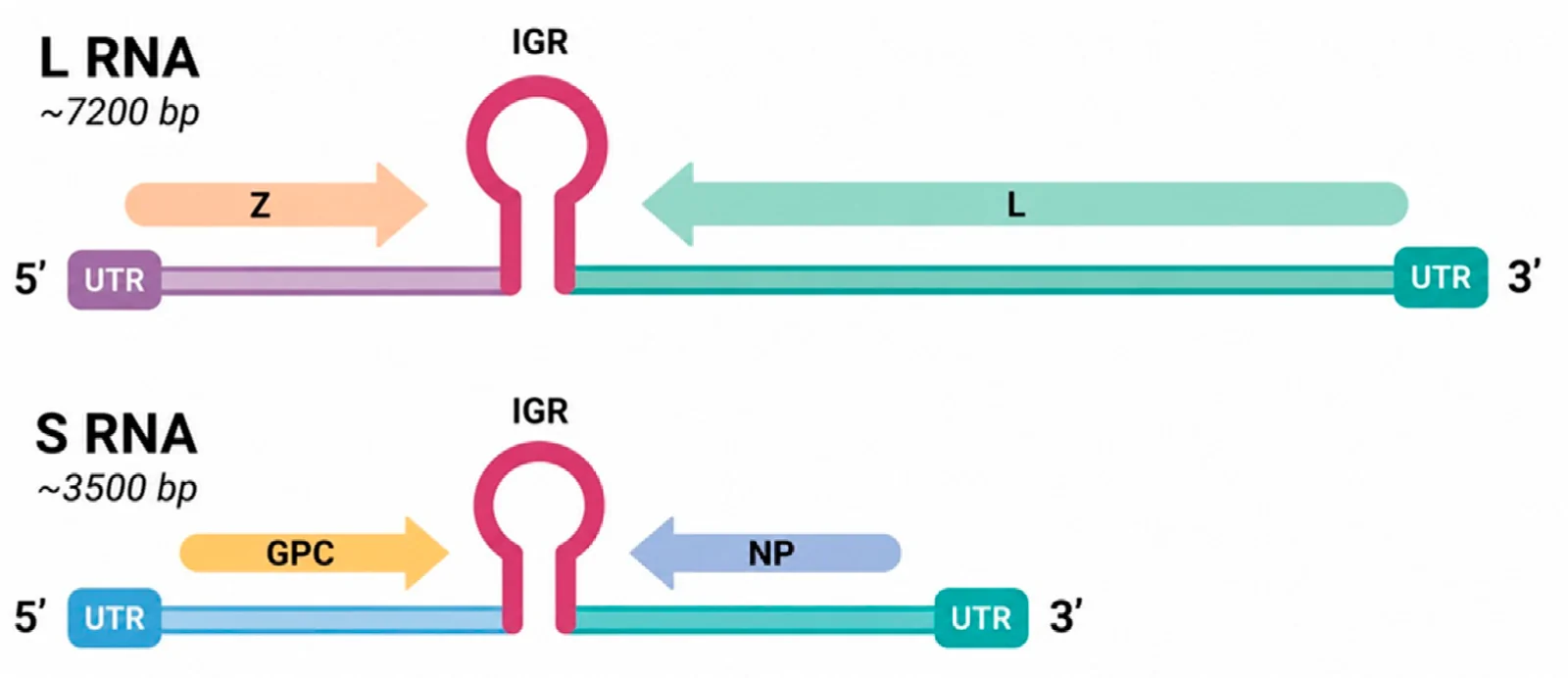
Schematic representation of mammarenavirus genome organization. Illustration was created by using Inkscape, version 1.4.3.

The mammarenaviruses genome is bisegmented, consisting of a Large (L) segment (~7.2 kb) and a Small (S) segment (~3.5 kb), encoding the zinc-binding matrix protein (Z) and the viral RNA-dependent RNA polymerase (L) and a Small (S) segment (~3.5 kb) that encodes the envelope GP pre-cursor. Both segments present conserved reverse-complementary untranslated regions (UTRs) of 19 to 30 nucleotides at their 5′ and 3′ ends, promoting the formation of non-covalently closed “panhandle” structures that serve as replication promoters [23,24,25]. Using an ambisense coding strategy, each segment encodes two non-overlapping genes in opposite orientations, separated by highly structured intergenic regions (IGRs) that fold into stable hairpin loops to terminate transcription (Figure 2).

## 4. Epidemiology and Host Reservoirs

Based on their geographic distribution, antigenic and phylogenetic relationships, mammarenaviruses are splitted into Old World (OW) and New World (NW) arenaviruses, also known as the LASV-LCMV and the TCRV serocomplex, respectively [2,3].

The members of the OW group are endemic in the Eastern Hemisphere, while the NW in the Western Hemisphere. The OW and NW groups are polyphyletic and contain both human-pathogenic and non-pathogenic viral strains, comprising more than 50 species along with recently discovered, unclassified mammarenaviruses found in bats, hedgehogs, and pikas [5,13,26]. The OW group includes the prototypic LASV and LCMV, as well as the newly emerged LUJV. LASV causes Lassa fever (LF), a disease with significant mortality that is highly prevalent in West Africa, especially Nigeria, Liberia, Guinea, and Sierra Leone. Recent computational models estimate that LASV infects more than 900,000 people each year [27,28]. LF is listed by the World Health Organization (WHO) as one of the diseases posing the greatest risk to public health due to its epidemic potential and the absence of effective countermeasures [29]. The second zoonotic African OW arenavirus, LUJV, was identified in 2008 following an outbreak of human hemorrhagic fever in South Africa, which was imported from Zambia [30]. Although LUJV was associated with high case fatality rates in humans, its specific host range and geographical distribution remain unknown [30,31]. While the OW viruses described above are generally geographically confined to the African continent, LCMV circulates globally, making it a public health concern on every continent except Antarctica [32].

The clinical spectrum of human LCMV infection is highly variable, ranging from mild, self-limiting febrile illness to severe neurological diseases, including meningitis and encephalitis. Furthermore, infection during pregnancy is associated with severe teratogenic outcomes, such as congenital hydrocephalus and chorioretinitis [32,33]. Seroprevalence studies in the general population have reported LCMV antibody prevalence rates of up to 15%. Nevertheless, due to its nonspecific clinical manifestations, limited physician awareness, suboptimal diagnostic tools, and inconsistent reporting, LCMV remains a largely underrecognized public health threat [32].

NW arenaviruses are geographically restricted to the Americas and include several highly pathogenic agents responsible for severe hemorrhagic fevers [18]. These include JUNV, the causative agent of Argentine Hemorrhagic Fever [34]; MACV and CHAPV viruses, causing Bolivian and Chapare Hemorrhagic Fevers [35,36]; GTOV, responsible for Venezuelan Hemorrhagic Fever [37]; and SABV, the agent of Brazilian Hemorrhagic Fever [38]. While the epidemiology of Argentine Haemorrhagic Fever is well-documented, the prevalence rate of the other South American diseases remains largely unknown [2]. Among the zoonotic NW arenaviruses, Whitewater Arroyo Virus represents a North American mammarenavirus of potential zoonotic concern that has also been linked to fatal hemorrhagic illnesses in the United States [39,40]. The primary reservoirs of most known mammarenaviruses are rodents of the superfamily Muroidea [1]. In particular, the reservoir hosts for OW mammarenaviruses are mainly members of the subfamily Murinae in the family Muridae [41,42]. In West Africa, the endemic circulation of LASV is sustained by the Natal multimammate mouse, *Mastomys natalensis* Smith, 1834 (*M. natalensis*) [43]. These commensal animals are one of the predominant rodent species throughout Sub-Saharan Africa [44] and are usually found close to rural human dwellings [45,46].

The epidemiology of LASV exhibits a profound seasonal rhythm correlated with macroclimate shifts and landscape management practices. During the dry season, widespread agricultural bush-burning and the subsequent depletion of wild foraging matrices compel *M. natalensis* populations to undergo massive domestic migrations, significantly accelerating the viral contamination of domestic food storehouses and water supplies [47]. In contrast to the tropical, localized endemicity of LASV, LCMV manifests a cosmopolitan distribution reflecting the widespread presence of its primary reservoir, the common house mouse, *M. musculus* [32].

NW arenaviruses are predominantly associated with rodents of the family Cricetidae, with the subfamily Sigmodontinae acting as the primary reservoirs in South America and Neotominae in North America [35,42,48,49,50,51,52]. JUNV is thought to be maintained primarily in *Calomys musculinus* Thomas, 1913, the drylands vesper mouse or corn mouse, and *Calomys laucha* Fischer, 1814, the small vesper mouse [34]. *Zygodontomys brevicauda* (J. A. Allen & Chapman, 1893) the short-tailed cane mouse, appears to be a reservoir host for GTOV, although it is also found occasionally in *Sigmodon alstoni* Thomas, 1881 (Alston’s cotton rat) [53]. *Calomys callosus* Rengger,1830, the large vesper mouse, is known to carry MACV [54]. This mouse can also become chronically infected with JUNV after experimental inoculation [55]. Whitewater Arroyo virus has been found in *Neotoma albigula* Hartley, 1894 (white-throated wood rat), *Neotoma mexicana* Baird, 1855, *Neotoma micropus* Baird, 1855, and *Neotoma cinerea* Schreber, 1776 [48,49,56]. The natural hosts for CHAPV and SABV are not yet known, but they are assumed to be rodents as well [30,31,57]. An exception is the TCRV, isolated from *Artibeus* bats and mosquitoes in Trinidad and Tobago [6], and lone star ticks (*Amblyomma americanum*) in Florida [58]. Although evidence suggests its circulation among bats throughout tropical America, its reservoir and geographic range are still debated [58,59,60,61,62,63,64].

Mammarenaviruses typically establish asymptomatic infections in their natural reservoir hosts, marked by chronic viremia and prolonged shedding of high viral titers through all secreta and excreta [42]. Horizontally infected adult rodents usually clear the virus and mount lifelong immunity [65,66,67], whereas vertical transmission early in ontogeny often results in a lifelong persistent infection that is crucial to the maintenance of the virus in nature [65,66,67,68,69,70]. Humans primarily acquire infection through the inhalation of aerosolized droplets or dust particles contaminated with rodent urine or feces, as well as via the ingestion of contaminated food and water or direct contact between excreta/secreta and abraded skin [2]. These exposures typically occur when human activities overlap with the natural habitats of reservoir rodents. Additionally, the handling and consumption of rodent meat represents a notable route of transmission, particularly in low-resource communities reliant on bushmeat [71,72,73]. Transmission may also occur through rodent bites, although considered a less common source of exposure [74]. Horizontal human-to-human transmission is rare; however, it has been documented following organ transplantation [75,76,77] and within healthcare settings characterized by inadequate infection prevention and control practices [30,78,79,80]. Furthermore, vertical transmission of LCMV from infected pregnant women to their fetuses is well-established [81,82], whereas sexual transmission remains exceedingly rare [83]. Finally, Olayemi et al. [84] performed a phylogenetic analysis based on a combination of partial GP and NP sequences of LASV in West Africa, which revealed that human LASV strains are ancestral to those found in rodents. This suggests that human-to-rodent spillback might occur through environmental contamination, particularly when rodents are exposed to infectious human waste or sewage.

## 5. Newly Discovered Mammarenaviruses and Novel Hosts

Recent metagenomic and wildlife surveillance studies allowed the identification of several novel mammarenaviruses, expanding the known host range of the genus far beyond its traditional rodent limits (Table 1).

Originally identified in 2013 in the Wenzhou region of China, Wenzhou Virus (WENV, species *Mammarenavirus wenzhouense*) represents the first evidence of cross-order mammarenavirus [12]. Li et al. [12], by molecular testing 351 rodents for mammarenaviral infections, found viral RNA in a total of 42 samples collected from rodents of the genus *Rattus* and *Niviventer* (12.0%), and from Asian house shrews (*Suncus murinus* Linnaeus, 1766, *S. murinus*) (4.4%) belonging to the order Eulipotyphla. Considering the complete genome sequencing, the new virus clustered with other known OW mammarenaviruses. Experimental WENV infection reveals strong systemic tissue tropism, with viral RNA recovered from fecal samples and from the liver, lungs, heart, kidneys, and spleen of rodents or shrews [12]. Subsequent studies in small mammals have demonstrated that WENV and several genetic variants are widely distributed across Asia, exhibiting an exceptionally broad host range, infecting multiple *Rattus* species, *M. musculus* and *Apodemus agrarius* Pallas, 1711 [87,88,89,90,91,92,93,94]. However, due to the significantly lower viral prevalence and lack of longitudinal shedding data in *S. murinus*, the ecological role of shrews in WENV epidemiology remains uncertain. Interestingly, following the identification of the WENV genetic variant, named Cardamones, in peridomestic brown rats (*Rattus norvegicus* Berkenhout, 1769) and Pacific rats (*Rattus exulans* Peale, 1848) in Cambodia, Blasdell et al. [95] analyzed human clinical samples to establish the potential for zoonotic transmission and if the infection is associated with clinical disease [95]. Using a WENV IgG enzyme-linked immunosorbent assay, seropositivity was detected in 17.4% of patients presenting with flu-like symptoms but tested negative for dengue and influenza infections, and in 13.2% of healthy individuals, indicating human exposure to WENV or closely related mammarenaviruses. In addition, viral RNA was detected in the respiratory specimens of 0.6% of Cambodian patients presenting with mild or severe respiratory symptoms. However, a definitive causal link could not be established due to the mild and non-specific symptoms, the presence of co-infections with other respiratory pathogens in half of the positive patients, and the small sample size [95]. In a subsequent study, Guo et al. [96] investigating 830 serum samples from healthy individuals (aged 0–70 years) in two Chinese provinces found seroprevalences of 1.5% in children and 4.6% in adults. Compared with findings from Southeastern Asia [95], these lower rates were attributed to the higher specificity of the detection method, as well as to geographical or temporal differences [96]. All these findings raise awareness about this emerging virus, encouraging further studies.

The discovery of Jerboa arenavirus (JEAreV), subsequently classified in the species *Mammarenavirus alashanense* (Alxa virus) provided the first evidence of host-range expansion from traditional muroid families to non-muroid rodents (family Dipodidae), highlighting that mammarenavirus divergence can occur within isolated ecological niches. Identified from the three-toed jerboa (*Dipus sagitta* Pallas, 1773), in 2014 in Inner Mongolia (China), this novel mammarenavirus exhibited an overall prevalence of 8.0% and strict host specificity, remaining absent in sympatric jerboa species such as *Euchoreutes naso* Sclater, 1891, and *Allactaga sibirica* Forster, 1778. Phylogenetic analyses based on the L and S segments placed JEAreV within the OW complex but on highly independent branches [18]. This distinct topological position, coupled with the absence of alternative rodent hosts in the environment, suggests that JEAreV evolved separately within desert rodent niches as a stable, species-specific resident rather than an accidental spillover from sympatric muroid rodents [11].

The paradigm of mammarenaviruses as strictly rodent-borne pathogens has been further challenged by discoveries in other Eulipotyphla beyond shrews. Robust evidence of mammarenavirus circulation in this order has emerged from studies on hedgehogs (Family Erinaceidae). Specifically, the detection of the highly divergent Mecsek Mountains Virus (MEMV) in Northern white-breasted hedgehogs (*Erinaceus roumanicus* Barrett-Hamilton, 1900) in Hungary marked the first confirmed association of arenaviruses with erinaceids [13]. Although the virus showed high genetic relatedness with Alxa virus [11], pairwise sequence identities were below the ICTV demarcation cut off values [1], leading to its classification as a novel species, *Mammarenavirus mecsekense*, within the OW group [13]. In 2025, Takáts et al. [86] screened additional faecal specimens collected from Northern white-breasted hedgehogs across different sampling areas in Hungary, characterizing five novel mammarenavirus strains. Although their closest relative was the known OW MEMV [13], sequence and phylogenetic analyses showed that these new strains possess unique genome motifs, likely representing a second novel species, tentatively named Pannonia mammarenavirus (PANV) [86]. Consequently, *Erinaceus roumanicus* in Hungary hosts at least two different mammarenavirus [86]. Parallel investigations indicated that the relationship between hedgehogs and mammarenaviruses extends beyond a single country, revealing a complex viral co-circulation across Europe. In European Russia, Lukina-Gronskaya et al. (2024) [85] identified highly variable strains in oral and anal swabs from *Erinaceus* species, collectively designated as Erinaceus Arenaviruses (EriAreV). Despite grouping into the hedgehogs mammarenavirus clade, their high genetic diversity warrants classification as another distinct species [85]. In 2025, the identification of an additional mammarenavirus in liver and duodenal samples of necropsied European hedgehogs (*Erinaceus europaeus* Linnaeus, 1758) collected in Italy was also reported [14]. On pairwise sequence comparison, the four obtained Erinaceus Europaeus Arenaviruses (EEAVs) were more closely related to the white-breasted hedgehog MEMV [13], but according to the species criteria were to be considered a separate species [14]. As reported in the study of Takáts et al. (2025), S and L segments phylogenetic analyses of hedgehog sequences from Hungary [13,86], Russia [85] and Italy [14], including two Chinese hedgehog (*Erinaceus amurensis* Schrenk, 1859) sequences from databases (OP899820–OP899821, unpublished data), showed that all strains were grouped in an independent clade, within the OW complex, alongside the MEMV and Alxa Virus [86]. Within this clade, hedgehog mammarenavirus sequences were clustered by geographic origin, representing at least five potentially distinct hedgehog-related mammarenavirus species [86].

All these findings confirm that erinaceids are not accidental spillover hosts, but they could represent species-specific evolutionary reservoirs for multiple distinct mammarenavirus species. The reservoir status could also be supported by the detection of viral RNA, in addition to duodenum and liver, in multiple organs, including the brain, spleen, kidney, and lungs, of the positive Italian *Erinaceus europaeus*, which suggests a systemic infection despite the lack of associated histopathological lesions [14]. Moreover, the identification of positive Russian hedgehogs among both active and captive hibernating individuals, kept under strict, long-term isolation, demonstrated the virus’s long-term persistence [85]. Taken together, these findings are highly consistent with the ability of arenaviruses to persist in hedgehogs, closely mirroring the chronic, asymptomatic shedding dynamics typically observed in their natural rodent host [97].

Finally, the discovery of Plateau Pika Virus (PPV, species *Mammarenavirus batangense*) by Luo et al. [5] in plateau pikas (*Ochotona curzoniae* Hodgson, 1858) on the Qinghai–Tibet Plateau represents the first identification of a mammarenavirus in the order Lagomorpha. PPV RNA was detected in 6.3% of intestinal samples, and high viral loads also in internal organs, as lungs, kidneys, heart, liver, and spleen, established pikas as natural hosts of PPV [5]. Although PPV displayed the typical genomic organization, phylogenetic analyses demonstrated that it is highly divergent from both OW and NW mammarenaviruses, potentially representing an ancient lineage that diverged from classical mammarenaviruses approximately 77 to 88 million years ago, thereby far preceding the estimated divergence of classical OW and NW groups [5]. In the same study, by linking wildlife surveillance directly to human clinical risk, a retrospective serological survey in the high-altitude Qinghai–Tibet region revealed a seropositivity rate of 2.4% among human patients [5]. Coupled with in vitro evidence showing that PPV efficiently infects and replicates in human and non-human primate cell lines, these findings provided preliminary clues to the possibility of cross-species transmission to humans [5,98].

## 6. Discussion

The identification of novel arenaviruses and new hosts highlights that the true diversity of the *Mammarenavirus* genus remains largely unknown, likely extending to many understudied mammalian species. Prior to the discovery of MEMV in Hungary [13], the epidemiological distribution of mammarenaviruses in Europe was believed to be restricted to a single circulating species, the cosmopolitan LCMV [32]. The subsequent identification of a complex, geographically clustered network of distinct mammarenavirus species endemic to erinaceids across the continent, including PANV in Hungary, EriAreV in Russia [85], and EEAV in Italy [14], has reshaped our understanding of mammarenavirus, host co-evolution and, consequently, their epidemiology on the European continent [86]. Importantly, rather than representing accidental, transient spillover events from sympatric rodents, the systemic tissue tropism and long-term viral persistence observed either in necropsied or in hibernating hedgehogs strongly suggest that these insectivores serve as stable, species-specific evolutionary reservoirs [14,85].

The expansion of the host range into non-rodent small mammals, including other eulipotyphla such as shrews [12] and members of the order Lagomorpha such as pikas [5], may carry critical public health implications. For instance, significant seroprevalence rates and the detection of viral RNA in human respiratory specimens have been linked to WENV exposures in Southeastern Asia [95,96]. Similarly, retrospective serological surveys have demonstrated human exposure to the newly discovered PPV [5]. While systemic replication points to a reservoir status of plateau pikas, the absolute risk of zoonotic spillover remains speculative and needs to be further investigated [5,98].

In addition, besides discovering new mammarenaviruses, recent research highlights the increasing detection of well-known viral species in unexpected animal hosts. A clear example is LCMV, historically associated almost exclusively with its reservoir *M. musculus* [32]. However, viral RNA and antibodies have also been detected in other rodent species [99,100,101,102,103,104]. In a wild rodent survey in the United Kingdom [99], LCMV RNA and antibodies were detected in wood mice (*Apodemus sylvaticus* Linnaeus, 1758). Serological evidence was also found in *Sciurus vulgaris* Linnaeus, 1758, *Microtus agrestis* Linnaeus, 1761, *Micromys minutis* Pallas, 1771, even in captive-housed *Cynomys ludovicianus* Ord, 1815, and in *Rattus norvegicus* [99]. More recently, in a surveillance study conducted in Gabon [105], the viral RNA was detected in the rodent, *Lophuromys sikapusi* Temminck, 1853, and in the porcupine, *Atherurus africanus* J. E. Gray, 1842, while anti-LCMV-like antibodies were detected in three African pigmy mice (*Mus minutoides* Smith, 1834), in one African soft-furred mice (*Praomys missonei* Van der Straeten & Dieterlen, 1987) and in three Goliath shrews (*Crocidura goliath* Thomas, 1906) [105]. Similarly, reservoir expansion has been documented for LASV. Beyond the primary reservoir *M. natalensis* [43], LASV has been detected in other rodent species, including the closely related *Mastomys erythroleucus* Temminck, 1853 (Guinea multimammate mouse), and less commensal rodents such as *Mus baoulei* (Vermeiren & Verheyen, 1980), and *Hylomyscus* pamfi (Nicolas, Olayemi, Wendelen, & Colyn, 2010) [106,107]. Similarly, viruses of different natures were detected in mammals that were completely atypical for them [108,109]. In light of these findings, epidemiological investigations on mammarenaviruses should extend beyond rodents, their primary known reservoirs, to encompass alternative wildlife hosts with comparable depth.

## 7. Conclusions

In conclusion, the growing evidence of an expanding mammarenavirus host range raises concerns regarding their zoonotic potential, particularly considering that these viruses are already known to infect humans. Looking ahead, critical research gaps must be addressed to comprehensively evaluate the zoonotic and spillover risks associated with these emerging mammarenaviruses. Furthermore, expanding surveillance beyond traditional rodent populations is essential to identify novel reservoirs, elucidate the molecular mechanisms driving host adaptation, and fully understand the true evolutionary diversity of the genus *Mammarenavirus*. More specifically, a primary epidemiological priority is to definitively determine whether these newly identified non-rodent small mammal hosts act merely as incidental hosts or as competent natural reservoirs capable of sustaining intra-species transmission. Resolving this fundamental question will require long-term longitudinal ecological monitoring of wildlife populations in tandem with metagenomic screening of alternative hosts. Furthermore, the documented 2.4% seroprevalence of PPV-specific antibodies among humans in the Qinghai–Tibet Plateau underscores the need for expanded serological surveillance studies in human populations sharing habitats with these novel wildlife hosts. Ultimately, as anthropogenic environmental encroachment and climate-driven ecological shifts increasingly blur the interfaces between wildlife and human settlements [110,111], integrating molecular, ecological, and serological data within a cohesive One Health framework will be paramount to predicting, detecting, and mitigating the emergence of novel zoonotic mammarenaviruses.

## Figures and Tables

**Table 1:** Host range, sample types, and geographic distribution of novel mammarenaviruses in non-muroid species.

Viral Species	Virus Name	Non-Muroid Host	Order/Family	Sample Type	Geographic Area	Reference
*Mammarenavirus wenzhouense*	Wenzhou virus (WENV)	Asian house shrews (*Suncus murinus*)	Eulipotyphla/ Soricidae	Feces, heart, liver, spleen, lungs, kidneys	Wenzhou region of China	[12]
*Mammarenavirus alashanense*	Alxa virus	Three-toed jerboas (*Dipus sagitta*)	Rodentia/ Dipodidae	Anal swabs	Inner Mongolia, China	[11]
*Mammarenavirus mecsekense*	Mecsek Mountains virus (MEMV)	Northern white-breasted hedgehogs (*Erinaceus roumanicus*)	Eulipotyphla/ Erinaceidae	Feces	Hungary	[13]
*Mammarenavirus* *batangense*	Plateau Pika Virus (PPV)	Plateau pikas (*Ochotona curzoniae*)	Lagomorpha/ Ochotonidae	Feces	Yushu, Qinghai Province, China	[5]
Unclassified	Erinaceus Arenaviruses (EriAreV)	*Erinaceus* spp.	Eulipotyphla/ Erinaceidae	Oral and anal swabs	European Russia and Central Siberia	[85]
Unclassified	Erinaceus Europaeus Arenaviruses (EEAVs)	European hedgehogs (*Erinaceus europaeus*)	Eulipotyphla/Erinaceidae	Liver, duodenum	Northwestern Italy	[14]
Unclassified	Pannonia mammarenavirus (PANV)	Northern white-breasted hedgehogs (*Erinaceus roumanicus*)	Eulipotyphla/Erinaceidae	Feces	Hungary	[86]

## Data Availability

No new data were created or analyzed in this study. Data sharing is not applicable to this article.
